# Rationale of Inductive Evidence
*"An Essay upon the Philosophy of Evidence; with a Discussion concerning the Belief in Clairvoyance," by Watkin Williams, of the Inner Temple. Chapman, London. 1853.


**Published:** 1853-07-01

**Authors:** 


### Art. IV.—RATIONALE OF INDUCTIVE EVIDENCE.*

We live in the days of *clairvoyance*—which may be defined the art of seeing through "nine-inch boards," and a great deal thicker; for the clairvoyant tells of what is done in distant lands, sees sick persons through the whole diameter of the earth, for aught we know, and announces to their friends at home how they do; not to speak of minor and more proximate matters; such as discovering and describing stolen property and the thieves, seeing their own or others' insides, and what may be the matter with them—nay, what medicines are to cure them, though the doctors may have all failed as well as differed. The clairvoyant reads with fingers, forehead, or stomach; and, as the poet says, "by daylight, or candle-light, eyes being shut," sees through thick envelopes and closed book-covers; hears bells toll in the dead of night announcing that the souls of friends have passed into the invisible world, their deaths having occurred, it may be, many miles off; and the notification being quicker than even the arrangements of the electric telegraph. The clairvoyant, when put *en rapport*, will "take a walk," as it is called, with you, not only through streets and squares, but over hedge and ditch, mountain and valley, land and water; and if you are fortunate enough to have some mansion in the country, will tell you, though neither knowing your person nor your home, what fences are around your trees, iron or wooden, what armour hangs up in your hall, and how placed,—what grain your homestead is sown with, and a great many more such things equally extraordinary. Now all this is certainly very marvellous—that is to say, on the proviso of our brother Jonathan—"if true." And we are among those who would not be among the number to say we will not believe because all clairvoyants cannot, nor any, at all times, see the money in people's pockets, nor the numbers of £100 bank notes, sealed up in the banker's drawer, and only awaiting the skill of the fortunate clairvoyant to draw it forth from its depository, and to appropriate it as his or her own. We

* "An Essay upon the Philosophy of Evidence; with a Discussion concerning the Belief in Clairvoyance," by Watkin Williams, of the Inner Temple. Chapman, London. 1853.

would believe all this, and more, if necessary, when favoured with the proper sort of evidence, and enough of it.

Mr. Williams proposes first to discuss, in brief, the philosophy of evidence, and then to apply it to clairvoyance; not because he thinks that other illustrations might not be found equally to exemplify his principles, but simply because this happens to be a "popularly received system." We shall give to our readers some account of the general principles of the pamphlet, irrespectively, of course, in the first instance, of their application. We shall then be better prepared to understand what the author has to say of clairvoyance itself.

His opinion is, that among the theories overturned by the principles of his Treatise, is that of Reid and others, adopted by Taylor in his work on the Law of Evidence—"that children have an instinctive disposition to confide in the veracity of others, and that the mind is *not* naturally in equilibrio, without any inclination to the side of belief more than to that of disbelief." Mr. Williams maintains, on the other hand, that "the principle of our childish belief is plainly reducible to association of ideas; and that it is no more instinctive, than it is so to speak English, or to be a Catholic." From this preliminary doctrine, for it occurs in the Preface, we are constrained to dissent, so far at least as it seems to deny any innate tendency to confide in testimony. Granted that association has to do with our belief, at all periods of life: in all cases which are properly to be called those of belief, there is no doubt an association of antecedents and consequents. But whence the tendency to this association? Why, because the sign is presented as representing the thing signified, do we expect them to be united? In other words, what is the foundation of the principle of association? Surely the natural (instinctive) constitution of the human mind. In the case of very young children, there is evidently no such belief as that which is the result of weighing antagonist evidences; all seems blind, spontaneous and impulsive. We see no objection to saying that the instinctive or natural tendency to regard the sign as the representative of the thing signified or the reality, may make children of a certain age more ready to believe what is told them than adults are; in other words, association has to do with their belief. But the natural tendency to expect that what is affirmed in words is true in fact, appears to us only to be checked by subsequent experience, in the course of which we learn that all that is said is not true. The case of learning English does not meet the question: it is a case of the mere arbitrary connexion of certain objects and ends with certain sounds; it is purely a case of naming. There is no natural tendency to learn English, any more than any other language, or to learn catholic doctrines more than others; but it does not follow from this, that when sounds which may be uttered in any language that has been learned are made use of in affirmation or denial, there is no tendency in the simple, unsophisticated mind of childhood to expect things to agree with either of these predications rather than the other, until experience has taught not only the meaning of the words, but their agreement with events. We strongly suspect that the reverse is the case—that there is a natural tendency to confide in testimony, until experience teaches us to restrain and correct it.

Our author adopts a usual distinction which is followed in regard to evidence and belief, between "mathematical" (logical) truths, and "matters of fact." That there should be a triangle, two sides of which are not greater than the third side, is a logical impossibility, and may be proved so by the *reductio ad absurdum*, or Leibnitz's principle of contradiction; by which, and the principle of identity, which comes to the same thing in reality, all logical propositions may be tested. The contrary of mathematical truths, for instance, is not only false but inconceivable. Matters of fact no doubt stand on a different basis. While it is not only true but incapable of being conceived of as false, that two right lines cannot enclose a space,—it is true that Napoleon the First was a real personage, but it is quite conceivable that he might have been a fictitious one, as is ingeniously assumed and argued in Archbishop Whately's "Historic Doubts," a masterly though a very quiet thrust at the heart's core of Hume's scepticism. The example given by our author respecting ice (any other material would have done as well) not being capable of bearing a weight, without breaking, greater than its own actual strength, is doubtless, as he remarks, as much a mathematical (or logical) truth as the one concerning the triangle. It is found that any material whatever has its attraction of cohesion overcome whenever a force greater than that attraction is in antagonism with it, and, of course, it would amount to a contradiction to suppose that an actually existing force somewhat less than another is equal to it.

In order to illustrate the evidence of *matters of fact*, Mr. Williams takes an example from our knowledge of the properties of familiar objects. I see a white flaky substance falling from the sky in winter: and though I do not *feel* it, I am firmly persuaded that it is cold; and, therefore, I call it *snow*. "I believe it to be cold, because in every *other* instance of which I have had personal experience, of which I have heard, or of which I have read, I have found that a body having the same appearance, and the same source, and coming under similar circumstances, has been cold: from this I am led to believe that this substance will also be found to be cold. There is, in fact, in my mind, an inseparable connexion between the idea of a white flaky substance falling from the clouds, and the idea that it also possesses the tangible quality, cold."

On the former part of this solution we have nothing to remark: no doubt we expect the laws of nature to be as they have been; and because, so far as we know, this white falling substance has always been cold, we believe this which we are now looking at through the windows to be cold too, and that we should feel it to be so if we went out of doors and took a handful of it up. We believe this snow to be cold, on exactly the same principle that we believe that if a new species of horned animal should chance to be discovered, it will be found to have cloven feet. We believe it in fact, if we believe it intelligently, from induction. We say *intelligently*, for we would distinguish this intelligent belief from the mere association, which in the latter part of our quotation seems to be given as the solution, as though it were identical with the former. Now, we think that the bare fact that we can hardly see this white flaky substance falling, without also thinking of it as cold, (that is, that it has this as well as the other properties of snow) is not sufficient to exhaust the phenomenon of our deliberate, intelligent belief. There are instances in which we cannot help certain associations, and yet cases exist of men believing something to which their associations do not lead, but rather the contrary. No man can help seeing, or even thinking of colour but as though spread on a surface; and we seem to see extension by the eye, and not only to feel it by the muscles. Yet, there are many acute and observant thinkers (we do not now discuss their theory) who *believe*, in opposition to this psychological phenomenon, that we no more see extension by the eye than we see solidity or real distance by that organ. In short, they believe that our cognizance of extension by the eye is nothing more than one of the "acquired perceptions of sight;" in other words, they believe that by the eye we see nothing but colour, and that our supposing that we see extension is only a case of very high, but here delusive association. Dr. Thomas Brown, and Mr. James Mill, are examples, among many more, of this belief. In still more obvious cases, our intelligent conviction (for this is what belief ought to mean) is in direct opposition to the first impulses of thought produced by association. We are so accustomed to associate change of angular position in the objects we look at, with motion—that we cannot see this change of their position without also thinking of their motion. When we are sailing out from a sea-port, the town, and all the neighbouring scenery, appears a moving panorama; but do we believe that the apparent motion is real? We would, then, submit that our intelligent conviction that the white falling flakes are *cold* (we must not here say—that they are *snow*, which would be merely to say *snow* is *snow*), is not merely because we always think of *cold* when we see them, but because we have experienced, and know that they have always been cold, whenever felt.

Our author does not, however, appear to lay so much stress on the bare fact of association, as blending itself with our whole psychological state when we pronounce on some phenomenon of nature, (*e.g.* the unfelt cold of snow), as to prevent him from ultimately admitting, as he appears to do, and as he certainly ought to do, that in the anticipation of the given quality, before we have actually experienced it in the particular case, our conviction amounts to an inductive process of mind. We will, at least, assume that this is his opinion, as he says shortly after the example of the snow—"I think a little reflection will convince any one that the process by which the mind proceeds from *induction* to form a judgment concerning any matter of fact, is totally distinct from that which is expressed by syllogism."

In regard to the nature of "induction" as related to the evidence of matters of fact, it may be well to remark, that even those philosophers who have gone the farthest in their endeavours to reduce belief to *association*, have still admitted, as an essential ingredient in the evidence, a recognition of the uniformity of the laws of nature. Mr. James Mill, in his chapter on Belief, in the "Analysis of the Phenomena of the Human Mind," says: "I believe that the stranger who now passes before my window had a father and mother, was once an infant, then a boy, next a youth, then a man; and that he has been nourished by food from his birth; all this, from my belief in the uniformity of the laws of nature." And may it not with equal propriety be said, that this same confidence which we have in the uniformity of these laws, is the reason why we believe that wherever we see the white falling flakes of the substance we call snow, they are *cold?* Do we believe this simply from the fact that we cannot see these flakes without *thinking* of the cold which we have always found in conjunction with them? Is it not ultimately because we believe that this conjunction is not a casual, but a uniform phenomenon—the result of a natural law? Is not the inference that we draw, in any particular case, from our persuasion of such a law, an inference from induction, however rapidly it may be drawn?

It is necessary that we should here advert to a distinction which is, we will venture to suggest, in danger of being too much neglected in the analysis of psychological phenomena. It appears to us to have been overlooked to a great extent, in the philosophical investigation of the point now before us, the rationale of evidence and belief. In all mental analysis we should, as carefully as we may, distinguish between the chronological development of the phenomena, and their logical content when once developed. Now, we think that what we commonly call *belief*, in a very young child, is not the belief of intelligence and the growth of reason. We are ready to grant the young child believes that fire will burn him when he has once found it doing so, and this on the same principle, that a dog believes that a stick which has hurt him once when beaten with it will hurt him again. We, no doubt, see here a case of association, and nothing seems to present itself in the phenomenon but the sign as recalling the thing signified, and with the latter the belief, such as it is. We may admit that so far the phenomenon is not at all logical. But the belief of manhood, in regard to facts and anticipated events has surely more of thought and reflection in it. Witness the very instance adduced by our author; we have always experienced a certain body (snow) having other qualities, also to be cold; we have heard and read that a similar experience has been that of mankind, so far as ever we are able to test that experience—very true: but why do we, after this reflection on the past and comparison of it with the present, conclude that the white flakes are *cold*, unless it be that by some means we have confidence in the uniformity of the laws of nature, and that the present is, and the future will be, under similar circumstances, as the past? We admit that in very young children, and in those animals which seem capable of the influence of association, as well as of bare mechanical instinct, the presence of the sign is enough for it at once to identify itself with the thing signified; but we are now speaking of the process of belief, as blended with a distinct growth of the rational faculty. We have "experienced" so and so, says our author—we have always "heard" it—we have "read" it—we think of "similar circumstances"—we are "led to believe."

Now this is no doubt a rapid process of induction; and we must again quote our author's remark: "I think a little reflection will convince any one that the process by which the mind proceeds from *induction* to form a judgment concerning any matter of fact, is totally distinct from that which is expressed by the syllogism."

Now the term *induction* is ambiguous. We have, from Aristotle downwards, a strictly inductive syllogism; but it is the counterpart of the deductive, and has its own laws. Aristotle, the great logician, remarks: "We believe all things either through the syllogism or from induction, which he also calls the syllogism from induction." (συλλογισμος εξ επαγωγης. *Analyt. Prior.* ii. 23.) We may thus exhibit their contrast:—

| Inductive. | Deductive. |
|---|---|
| x y z are A. | B is A. |
| x y z are (whole) B. | x y z are B. |
| B is A. | x y z are A. |

It is evident that the two are each the reverse of the other. In the above inductive syllogism we have an inference of the universal from the particular, all the particulars being actually enumerated; so that the conclusion is necessary by the formal laws of thought. In the latter, we have the particular inferred by the same laws from the universal. From a comparison of the two, it is clear that the conclusions can never be equally apodictical or necessary, unless every one of the particulars constituting B are enumerated. This alone is properly inductive demonstration.

But this is not the induction of the *Novum Organon* of Bacon, and of the natural sciences generally. We have not in these the separate inference of all the particulars. All we can attain to is the inference of the universal from the singular or particular, by *analogy*, or special presumption, founded on the object matter of some science, and legitimated as far as our experience reaches. *Ox, sheep, goat, ruminate;* they are horned animals; therefore, all *horned animals ruminate.* It must be admitted that the conclusion, here, however warranted by the material probability attaching to the facts of natural history, is not strictly logical, as not being absolutely necessitated by the self-evident and irreducible laws of thought. The conclusion, if we take the premises as they stand, is not sustained by them, either by the ordinary (deductive) syllogism, or by the "syllogism from induction:" for in the former case, if we say ABC, is D,

ABC, is E,

All E is D, we fall at once into *illicit process* of the minor term. In the latter case, we cannot say that A B C is (whole) E, and that, therefore, E is A B C, hence as before, we cannot conclude that E is necessarily D. Nevertheless, the *imperfect* induction—necessarily imperfect, because all the particular cases are not enumerated—of the above example respecting ruminant animals, is the type of innumerable other instances which exhibit the triumphs of modern science, by means of what is usually termed the "inductive method." The fact is, that when several particular cases (and no rule can be laid down for the number) which are analogous have been ascertained by actual observation, and have been stored in the memory, reason—the faculty of discerning relations, applies to the series of analogous observations the *à priori* principle that the laws of nature are constant; and what was found true by observation in a certain number of cases, becomes, by the application of this principle, a general law. The mind, by a certain tact of its own, arrives at consequences which transcend our actual observations, and anticipate them. The characteristic of this method is that it proceeds from certain results, obtained by actual observation, to a general principle in which they are included, and which is adopted as including all other cases of the like kind. How many cases are required, and under what circumstances, must depend on considerations drawn from the particular science or department of knowledge to which the cases belong. No general rule can be given. A few cases of horned animals being found with cloven feet might hardly raise a suspicion that the two always went together; but as observation of the twofold phenomena extended, and the facts became numerous, it would be thought probable that this law of nature was universal. It is now regarded as truth; and the naturalist and the palæontologist rely upon the anticipation that whatever animal may hereafter be found having horns, whether alive or in the fossil state, it will also have cloven feet. Yet as the induction can never be complete by an enumeration of all these animals, we cannot arrive at absolute demonstration as in the strictly formal logic; we can only attain to a very high probability or to moral certainty, which is by far the most common and the most useful guide which we have in all practical affairs.

We may, if we please, use the expression "*the inductive logic*," now so common, to distinguish the inductive inference as depending on only a partial survey of instances, from the inference which is formally demonstrative, but we regard it as an error to maintain that the two are *opposed* to each other, as is sometimes assumed. Still, we admit the distinction between the absolutely demonstrative, and the probable; and it is the probable, in whatever degree, that we really reach, and no more, unless we can find some law necessarily connecting the particular instances or the species, so that the connexion when observed, of some cases, may be inferred absolutely of all. Of this, mathematics contains many examples, but we must not look for them elsewhere. Mathematics furnishes means of showing that, in cases without number (series), if several terms all indicate a particular law, this law must be true of the next term, and of the next, and so on *in infinitum*. Such is the proof, known to all who have the slightest tincture of mathematics, that the *square of any number is equal to the sum of as many consecutive odd numbers, beginning with unity, as there are units in that number*.

It is evident that no little misunderstanding has arisen on the subject of induction from its ambiguity. Omitting the use of the term in the science of electricity, and perhaps elsewhere, as only signifying transference or accumulation, or at all events having no reference to any process of reasoning, the term is employed in a threefold sense. It is sometimes used merely for the objective business of collecting facts, *bringing* in or together a certain number of instances. At other times it means a formal inference of the universal from the particular as legitimated by the very laws of thought, as we have seen above. Again: (and this is its proper signification in all reasonings connected with the inductive sciences) it signifies an inference of the universal from the singular, by analogy.

Now we so far concede to our author, in reference to the nature of induction, that, if we understand by it an inquiry respecting individuals or species, in the matter of agreement or disagreement, this is not a logical process; it is a process which no doubt requires very accurate observation, and great judgment and caution: it is a process of inquiry into the question how far you are entitled to assert that what is true of individuals is true of species, or what is true of species is true of genus; and no particular rule can be laid down for thus framing your premiss. The number of examples previously necessary, and all the preliminary circumstances, may vary greatly in various sciences. A smaller number of examples may lead to the general conclusion that all iron ore of a certain kind (the magnet) attracts iron, than those which have induced the conclusion that quadrupeds deficient in upper cutting-teeth, ruminate. In order to form these conclusions, the observation and capacity of the physical philosopher and of the naturalist must first be employed, in order to ascertain when it may be pronounced that what belongs to the individuals which have been examined, may with a high degree of probability (perhaps amounting to moral certainty, though not to demonstration) be asserted to belong to the whole class, species, or genus, under which they fall. We admit, then, that the major premiss in this case is not a matter of formal logic, and possibly our author may regard this as a concession of the whole point, and that all reasoning from induction is an exemplification of nothing more than "association of ideas." We think not, however, for reasons above stated. Nor do we admit that when the major premiss is once determined on (which may be expressed by the general formula that *what belongs to certain individuals belongs to the class*) the "process by which the mind arrives at the conclusion is identical with that by which it arrives at the truth of the minor," as our author asserts.

For, to take an instance in which investigation of particulars is the only ground we have to rest on, and where there seems no reason to be given *à priori* for the connexion of the phenomena with each other: the naturalist has examined horned quadrupeds of various kinds and from various regions of the globe, and has found that they have all cloven feet. His skill and judgment are exercised in determining how far these quadrupeds are likely to resemble all other horned quadrupeds in the structure of their feet. He lays down a general rule (a new truth) by what is commonly termed the "Inductive process," and which ought always to be distinguished from the argument from Induction. No doubt the formation of this general principle is totally distinct from the syllogistic process. But our author says, that when the principle (major) is adopted, the minor and the conclusion are arrived at by the same process. The major being *all horned animals have cloven feet,* let us suppose the palæontologist to find some fossil bones. He at first discovers only those of the trunk, and he may not be certain to what species of quadruped they belong. None of the legs are found; but further research brings to light the head, and now it turns out that the animal is horned. By what process has this minor premiss been ascertained? clearly by nothing else than inspection. The animal has been actually found to belong to the class of horned quadrupeds; and whatever be the degree of probability that all the horned quadrupeds in the world have cloven feet, with that degree of probability alone can it be concluded that this horned quadruped has them. But is the process by which the mind arrives at the conclusion identical with that by which it arrives at the truth of the minor, as our author asserts? Certainly not. That A is B is laid down as a principle which may be depended on, as the result of observations and analogical indications: that C is A, is actually seen by inspection; but that C is B follows, not from inspection or investigation, but from the laws of thought. Grant the premises, having determined what weight the major premiss will bear, and the conclusion will be accordingly. In this case the conclusion is singular or individual. The same remarks, however, are applicable when the conclusion is universal; *e. g.*, that *quadrupeds* deficient in upper cutting-teeth ruminate, because *sheep, oxen, deer, and other animals thus deficient, ruminate*—this being the minor premiss, while the major is that *such a quality as this belonging to the individuals and kinds examined, belongs to the whole of animals of this sort.* Again, it is evident that this major premiss can only be sufficiently established by close observations and analogical considerations, apart from all logical forms: that the minor being admitted on inspection of the given quadrupeds (while in another view, and other relations, it may itself be regarded as a major premiss established like the former); and that the conclusion is not the result of observation, but follows from the laws of thought. In this latter example, of course, the strict order of the propositions is exactly inverted; but every tyro in logic knows that this does not in the slightest degree affect the argument, and no reader who happens to be not so much as even a tyro in logic, will find any controversy on the subject very edifying or intelligible.

It is evident, from the example with respect to *snow,* that our author would not see in the same light the process by which the mind works in believing (*i. e.*, coming to the conclusion) that any given fall of white flakes (snow) is cold. He refers our conclusion entirely to association. We have said quite enough to show that we dissent from him. We hold that the intelligent and deliberate conclusion, in any particular case, is just parallel with the above. We assume in our minds that a property (cold) which has been found in connexion, so many times, with certain other properties (whiteness, flakiness, etc.) under certain circumstances, will be again found attaching to the substance which possesses these properties: we then, by observation, are assured that what we are now looking at is white, flaky, etc., under exactly similar circumstances: we then conclude that this substance is also cold. Again, the minor premiss is by no means established on the same ground as the conclusion; the former resting (here) on ocular observation alone—the latter following by the mere laws of thought, regulated by the weight (degree of probability) of the major premiss. If we were to pursue the doctrine of the syllogism further, we should be repeating a great part of a former article, entitled, "The Theory of Reasoning," to which we beg to refer our readers.*

Having touched on the main points of the pamphlet, we have no space for the notice, to any extent, of other topics. We will, however, just add a word as to the dispute about cosmothetic materialism. We at first thought our author a Berkeleian; we soon found, however, that he is rather Kantian, on this point. He does not with Berkeley deny that matter is possible; he does not even deny its actual existence. He is rather a critical idealist than a material one, or as Kant would complimentarily say, a "fanatical idealist." Yet our author's language is often identical with that of Berkeley himself; for he says, "it is clear that the qualities which we actually perceive, are in reality nothing more than the sensations which we feel, and which we attribute to external objects operating upon the organs of the five senses. . . . . External objects, so far as we can have any knowledge concerning them, consist only of impressions and ideas produced upon our mind." From the whole tenor, however, of the author's remarks on this subject, it is evident that he came much nearer to the *noumenal* theory of Kant, than to the flat idealism of Berkeley.

But we must hasten to glance briefly at the second part of the work before us, in which the subject of *clairvoyance* comes under discussion. We quite agree with the author, that however plausible any theory of clairvoyance may be, it can derive no real support from anything but *facts;* and that, on the other hand, positive and sufficient testimony cannot be impugned by any *à priori* objections. We see these principles exemplified familiarly every day. No amount of witnesses to character can overthrow the actual proofs of a prisoner's guilt; and no degree of probability that he was the sort of man to do the action,

* See *Psychological Journal* for October, 1851.

can convict him, without direct proof. Yet, in the case of clairvoyance, "belief, on the one hand, and scepticism on the other, are constantly supported by the defensive theory, and *à priori* objections, respectively." We have then the detail of a narrative, told to the author by a friend of his, of a gentleman who, on a sudden, determined to call on his friend, who met him at the door, and told him that his dinner was nearly ready, for he knew of his coming through a clairvoyant lady who was in the house, though the gentleman had made up his mind to come only a few minutes before he started from home. The gentleman refused all belief, and his dinner, too, abruptly leaving the house. Of course, on the above principles, the gentleman was wrong. Our author, however, states, "if any one can be properly said to believe or disbelieve any proposition which he does not understand, I may be fairly said not to believe in clairvoyance." The reason is, he says, that he wants a more than ordinary amount of evidence. We can understand this; but we can hardly understand why he should say that he is called on to believe a proposition which he does not understand; for he is just called on to believe that a person has some means of knowing what is done many miles off, without being informed of it in any known way—or what is done in private, without being present.

Some very interesting matter follows, which we know not how to omit. Those instances are alluded to in which impressions are made on the brain, of which we have no immediate consciousness, (as often when a clock strikes) but which, still, we afterwards may remember. This is quite in harmony, to say the least of it, with the doctrine of an immaterial mind. On this principle, there are three distinct steps in our ordinary consciousness produced from without; an impression on some organ of sense, the perception of this impression by the brain, and the recognition of this impression by the mind in consciousness. Hence, a theory in support of clairvoyance. By whatever means the brain may receive on its fibres an impression, we may have consciousness of it, whether an impression be first made *ab extra*,—or not, as in dreams, in which we are conscious of impressions, as though from objects of sight, though these are not present. In imperfect sleep, a real impression on one organ will, by association, produce on the brain other and connected perceptions; witness the gentleman, in Edinburgh, who dreamed that the signal gun had been fired from the castle, and that the whole city was in commotion, in consequence of the news of Bonaparte's landing—and all this simply from the tongs falling down on the hearth. Let any perception be produced on the brain, by whatever means, and you have the corresponding thoughts in the mind. Conceive only, then, that the brain, in clairvoyance, is in an abnormal condition, and freed from the influence of the ordinary senses, and that it *becomes subject to the operation of a new sense,* which affects the brain as the external senses would, only that this new sense is of far wider sphere of action than they are, and is not obstructed by opaque objects—and you may have perceptions on the brain, cognized by consciousness, whatever be the distance, situation, or circumstances of the objects. This theory has been propounded in support of clairvoyance; but our author justly repeats that, after all, the question is one of fact; and if clairvoyance be a fact, it can be proved like other facts.

But how far is the theory plausible? "It is impossible," says our author, "that the mind can recognise in the objects perceived by this sense, the sources of any sensation producible upon the other senses, until this [sixth] sense has been used in conjunction with the other senses. The impressions perceived by this new sense cannot convey to the mind the ideas it is accustomed to perceive through the medium of the other senses." Of course the theoretic clairvoyant would say that this was nothing more than *begging the question.* But our author's objection here holds of his quasi-Berkleianism, in his language, at least; for he maintains that "all sensible qualities are nothing more than the impressions upon our ordinary senses." We hardly see how this language is quite consistent with the phenomena of dreaming to which he has alluded; and, for our parts, we would rather say that sensible qualities are the *causes* of our sensible impressions, than that they are the impressions themselves. Our author adds, "that this sixth sense must be different from the ordinary senses, and therefore its objects must be different," though they are supposed to be the same: and again, it follows that the knowledge obtained could not be expressed in the language of the five senses, and therefore could not be *communicated* by the clairvoyant. Now we can readily conceive that the theoretic clairvoyant would dispute this account of his theory. He might say that this sixth sense, power, or revelation, does *belong* to the objects of the senses, but is another mode of recognizing them. Our author himself gives something like another theory when he describes this supposed new faculty as "a sense emanating from the centre of sensation (the brain), and the sphere of whose action is decided by the will of some dominant spirit which guides it."

Take whichever of these aspects we may, however, of the theory of a manifestation of knowledge so extraordinary, we quite agree with the writer that "the most formidable objection still remains, namely, that there is not a shadow of evidence to support it [the theory], beyond the very facts which it is intended to support." Perhaps even the clairvoyants themselves would admit this; but then we know that they stoutly maintain the alleged facts. Hence we can only add, that time will, sooner or later, give opportunity for establishing the facts more convincingly than yet appears to be the case, to the bulk of scientific minds; or else time will finally set upon these alleged facts the seal of folly and delusion. We are quite content, in this age of wakefulness and inquiry, to await the result.

### Original Communications.

### HABIT—PHYSIOLOGICALLY CONSIDERED.

